# Gene expression signature predicts radiation sensitivity in cell lines using the integral of dose–response curve

**DOI:** 10.1186/s12885-023-11634-3

**Published:** 2024-01-02

**Authors:** Alona Kolnohuz, Leyla Ebrahimpour, Sevinj Yolchuyeva, Venkata S. K. Manem

**Affiliations:** 1 Quebec Heart & Lung Institute Research Center, Québec, Canada; 2 Department of Molecular Medicine, Laval University, Québec, Canada; 3 Department of Physics, Laval University, Québec, Canada; 4 Department of Mathematics and Computer Science, Université du Québec à Trois Rivières, Trois Rivières, Canada; 5 Centre de Recherche du CHU de Québec - Université Laval, Québec, Canada

**Keywords:** Radiation sensitivity, Radiotherapy, Dose-response, Gene expression signature, OMICS, Radiobiological model

## Abstract

**Background:**

Although substantial efforts have been made to build molecular biomarkers to predict radiation sensitivity, the ability to accurately stratify the patients is still limited. In this study, we aim to leverage large-scale radiogenomics datasets to build genomic predictors of radiation response using the *integral of the radiation dose–response curve*.

**Methods:**

Two radiogenomics datasets consisting of 511 and 60 cancer cell lines were utilized to develop genomic predictors of radiation sensitivity. The intrinsic radiation sensitivity, defined as the integral of the dose–response curve (AUC) was used as the radioresponse variable. The biological determinants driving AUC and SF2 were compared using pathway analysis. To build the predictive model, the largest and smallest datasets consisting of 511 and 60 cancer cell lines were used as the discovery and validation cohorts, respectively, with AUC as the response variable.

**Results:**

Utilizing a compendium of three pathway databases, we illustrated that integral of the radiobiological model provides a more comprehensive characterization of molecular processes underpinning radioresponse compared to SF2. Furthermore, more pathways were found to be unique to AUC than SF2—30, 288 and 38 in KEGG, REACTOME and WIKIPATHWAYS, respectively. Also, the leading-edge genes driving the biological pathways using AUC were unique and different compared to SF2. With regards to radiation sensitivity gene signature, we obtained a concordance index of 0.65 and 0.61 on the discovery and validation cohorts, respectively.

**Conclusion:**

We developed an integrated framework that quantifies the impact of physical radiation dose and the biological effect of radiation therapy in interventional pre-clinical model systems. With the availability of more data in the future, the clinical potential of this signature can be assessed, which will eventually provide a framework to integrate genomics into biologically-driven precision radiation oncology.

**Supplementary Information:**

The online version contains supplementary material available at 10.1186/s12885-023-11634-3.

## Introduction

Radiotherapy (RT) is among the most commonly used therapeutic modes of interventions in the management of cancer [1]. In routine clinical care, it is known that over half of all patients diagnosed with cancer undergo radiotherapy as a palliative and curative treatment modality in an adjuvant or a neoadjuvant setting [2]. Spanning over several decades, there have been numerous technological advancements in the way radiation is administered to patients for achieving a high therapeutic ratio [3–5]. For instance, image guidance techniques integrated with conformal radiotherapy and intensity-modulated radiotherapy indicated a superior therapeutic response for a variety of fractionation regimens [6]. For a longtime, the administration of radiotherapy in clinical practice has been guided by a tradeoff between the tumor control probability [7] and radiation-induced early- and late-toxicities [8, 9]. It is widely accepted that patients with similar stage, histology and anatomic features, known as the ‘one-size-fits-all’ dosing paradigm, is currently employed in routine clinical care. However, this ‘one-size-fits-all’ philosophy does not account for tumor biological features to design patient-specific radiation dosing regimens. Moreover, due to the inter-patient variability, patients treated with radiotherapy have a wide spectrum of clinical response [10]. Therefore, an in-depth understanding through the molecular lens is required, which has the potential to move RT into the realm of personalized radiation medicine based on patient-specific genomic profiles.

With the dawn of high throughput technologies, new research avenues have been opened to build biomarkers of treatment response using different types of OMICS data, such as, transcriptomics, proteomics, epigenomics, etc. This could help us to investigate the underlying biological mechanisms driving radiation response, thereby, enabling us to identify unique molecular targets accordingly. There have been several research efforts that were aimed towards the identification of genomic markers associated with radioresponse as well as to predict the risk of developing radiation-induced toxicities. One of the earliest works to predict radiation sensitivity was done by Eschrich et al., who developed a gene-based network using 48 cancer cell lines with SF2 (surviving fraction at 2Gy of radiation) as the radioresponse variable. Attempts to build radiation sensitivity gene signatures have continued to emanate over the years. A comprehensive overview of radiation response gene expression-based signatures can be found in a recently published work by Manem et al. [11]. In this compendium, all the molecular signatures of radiation sensitivity were built using the NCI-60 panel dataset with limited to no independent external validation raising concerns about their applicability and reproducibility. Moreover, these signatures showed little to no overlap among them. This can be attributed to the development of gene signatures arising from different technological platforms, various experimental assays used to generate dose–response profiles across labs, and statistical methods used to build them resulting in reproducibility issues, thereby, ultimately resulting in non-translatable clinical biomarkers. While on the contrary, a study led by Fan et al. found non-overlapping gene signatures to have high concordance, indicating common biological processes and redundancy among these signatures [6]. Considering these concerns with the development of biomarkers, it is crucial to apply robust statistical methods with independent discovery and validation cohorts before adopting to clinical practice. Through this manner, we can also avoid any spurious association of gene signatures with the biological determinants of radiation sensitivity.

Recently, Yard et al. profiled 511 cancer cell lines to different types of high-throughput screening—radiation sensitivity screen, while the same cell lines were also profiled at the transcriptomic level [12]. In their study, the authors found that radiation sensitivity is driven by the association between genomic instability and the alternations to DNA damage response. Given the concerns presented above along with the underlying biological complexity of the radiation response predictions, there is a dire need to combine the existing large-scale radiogenomics datasets and build robust multivariate genomic predictors and validate them on fully independent datasets. Till date, none of the studies in the literature have utilized these radiogenomics datasets to develop and validate radiation response biomarkers. Importantly, to build predictive biomarkers of RT, there is currently no consensus regarding the optimal response indicator for use across studies—AUC (Area under the curve of radiobiological model) or SF2 (surviving fraction of cells at 2Gy of RT). In this study, we establish that AUC is more robust radiation response predictor than SF2 to build molecular signatures. To the best of our knowledge, *this is the first time that both these large-scale radiogenomics datasets were analyzed in a single study with AUC under the radiobiological model as the response variable. This should provide us with adequate sample size to build molecular predictors and validate them in a fully independent dataset, i.e., discovery dataset* = *511 cancer cell lines and validation dataset* = *60 cancer cell lines*. Genomic predictors of radiation response built using preclinical data can be incorporated into the design of clinical trials, upon further external validation. This can potentially accelerate and translate genomically-driven radiation regimens to a clinical setting.

## Materials and methods

### Dose–response and gene expression data

We leveraged the largest radiogenomics dataset generated by Yard et al. (also termed as Cleveland data set (CL)) to build the transcriptomic signatures, while the smallest radiogenomics dataset published by Amundson et al. (also termed as NCI-60 (NCI)) was used to validate the gene signature. Within the CL dataset, the radiation sensitivity screening was performed across 511 cell lines comprising of 23 tissues. Multiple radiation doses of—1Gy, 2Gy, 3Gy, 4Gy, 6Gy, 8Gy, were administered to all the cell lines in the CL dataset to generate the dose–response data. The raw Illumina RNA-seq profiles of the CL dataset were retrieved from the CCLE website (http://www.broadinstitute.org/ccle/). The number of cell lines and tissues in the NCI cohort were 60 and 9, respectively. Only three radiation doses—2Gy, 4Gy, 6Gy, were administered to all the cell lines in the NCI cohort to obtain the cell viability data. The gene expression profiles for the NCI dataset were retrieved using the R package, *rcell miner*. This compendium of radiogenomics datasets were used to develop and validate gene signature for radiation sensitivity. Using our *RadioGx* platform [13], these datasets have earlier been processed. In the present study, the analyses were restricted to the genes common between the CL and NCI datasets, i.e., restricted for a total of 12,258 genes.

Through this radiation cell viability data, we can obtain the indicators of radiation response, which could be used to develop preclinical models. In the literature, radiation sensitivity was defined by two different indicators that can be extracted from the dose–response data, namely, area under the curve (AUC) of the fitted radiobiological model to the dose–response data and surviving fraction of cells at a specific dose level, 2 Gy (SF2). To this date, there is no consensus regarding the optimal indicator of radioresponse for use across studies. Although our earlier work has demonstrated that AUC captures more biological mechanisms than SF2, the comparison was done using only one pathway database. In this study, we generalized this concept, and utilized three pathway databases to compare the biological determinants of AUC and SF2.

### Radiobiological model

The relationship between the administered dose and the corresponding cell kill is given by the cell survival curve, which indicates the dose delivered against the number of surviving fraction of cells. The linear quadratic (LQ), also called the radiobiological model, is a formalism that is used to evaluate various clinically relevant radiation treatment regimens. The LQ model describes the fraction of cells that survived for a given radiation dose *D* (where, *D* can be an acute dose or a dose delivered in several fractions). The survival fraction of cells after radiotherapy is given by the following equation:

$$S = exp(-\alpha D-\beta {D^{2})}$$

where *D* is the total dose. In the above equation, the dimension of *α* is (1/Gy) that denotes the cellular radiosensitivity parameter, representing the direct action of lethal cell killing. And the dimension of *β* is (1/Gy^2^) that represents damage by DNA double-strand breaks. The ratio *α*/*β* depends on the tissue type. The value of *α*/*β* is high for tissues with early effects, and the linear value is crucial. For tissues with late effects, the value of *α*/*β* is low, and the quadratic term becomes important. Using the *RadioGx* platform, we fitted the radiation dose–response data to the LQ radiobiological model. AUC was then computed as the area under the curve of the fitted radiobiological model.

### Modeling approach

The integral under the dose–response curve was used as the radiation response indicator. For this analysis, we considered only those genes that were common between the two cohorts (12,258). The dimensionality of the features was further reduced by choosing all those genes that were associated with radiation response. For this purpose, we utilized our previously compiled compendium of radiation response gene signatures under oxic conditions [11], wherein, we curated a database of 35 gene expression signatures predictive of radiation response under both oxic and hypoxic conditions. These signatures have come from a variety of sources and encompass a number of derivation techniques (e.g., classification, regression, clustering, co-expression networks using gene expression data across different types of cancers). All of these signatures were developed to predict radiation sensitivity. We leveraged these genes that are predictive of radioresponse to decrease the dimensionality of features, which is also one of the common methodologies described in the literature. In total, there were 3402 unique genes related to radioresponse, out of which only 2836 genes were part of the gene expression data utilized in this study. Then, we employed the regression-based multivariate linear model to develop and validate the gene expression-based predictor of radiation sensitivity. The most significantly associated genes were selected based on the ranking of coefficient of correlation between gene expression and AUC, which were then used to fit a multivariate regression model. The performance of the model was evaluated using the concordance index metric, which is a generalization of the area under the ROC curve. The concordance index is defined as the proportion of all pairs of patients where one patient experienced the event of interest and the other patient did not experience the event, and the patient with the lower risk score was the one who did not experience the event. A concordance index of 0.5 represents a random predictor, while a concordance index of 1 denotes a perfect predictor. We used the implementation of the concordance index available in the *survcomp* package.

### Analysis framework

**Fig. 1:**
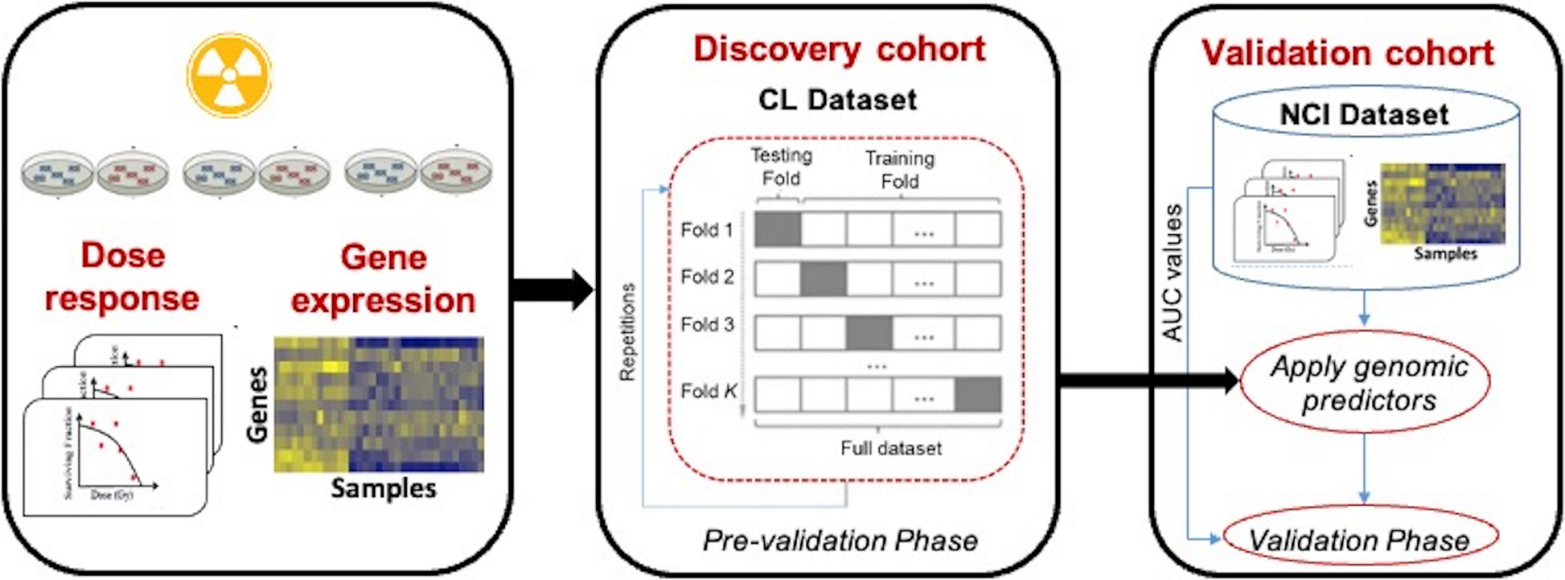
Analysis pipeline. Using the cross-validation framework in the discovery cohort, CL dataset, we carried out the pre-validation of genomic predictors for radiation sensitivity. Gene expression signature was developed using the full training set, which was then evaluated on a fully independent external cohort, NCI cohort

The framework for this study is presented in Fig. 1. The CL and NCI cohorts were used as discovery and validation datasets, respectively. Firstly, the pre-validation on the discovery dataset was performed, which consisted of 10 iterations/repetitions of 10-fold cross-validation. The model was then trained on the CL dataset and the NCI dataset was used as an independent validation cohort. The accuracy of the models was computed using the concordance index. By repeating ten times, we obtained the average accuracy for the model.

### Biological determinants of AUC and SF2

The pathway enrichment analysis was carried out using the gene set enrichment analysis (GSEA) methodology [14] with pathways defined by the C2 curated gene set from the MSIGDB (Molecular Signatures Database). For this study, we considered a compendium of three pathway databases, namely, ‘KEGG’, ‘REACTOME’ and ‘WIKIPATHWAYS’ consisting of 186, 1654 and 733 pathways, respectively. Firstly, the correlation was computed between SF2 and AUC with gene expression values. Then, genes were ranked based on their correlation coefficient. Using the GSEA methodology, we computed the enrichment score for each pathway along with statistical significance using a permutation test (1000 permutations). We performed the pathway analysis using the piano package [15]. For each pathway, the nominal p-values were corrected for multiple testing using the false discovery approach (FDR) method, with the *p.adjust* function in the base *R* package.

### Leading-edge gene analysis

In order to analyze if the same subset of genes were driving a pathway enriched by both AUC and SF2, we performed the leading-edge gene analysis. The GSEA methodology returns a subset of genes, termed as the leading-edge genes, which drives the enrichment statistic in the pathway analysis. The leading-edge genes were obtained from the enrichment score that is defined by the maximum deviation from zero. This set of leading-edge genes are considered to be of high biological interest due to appearing at higher frequencies among the pathway subsets, which can also be used to build gene signatures. We extracted the leading-edge genes from the pathways that were commonly enriched between the two radiation response indicators, AUC and SF2.

## Results

**Fig. 2:**
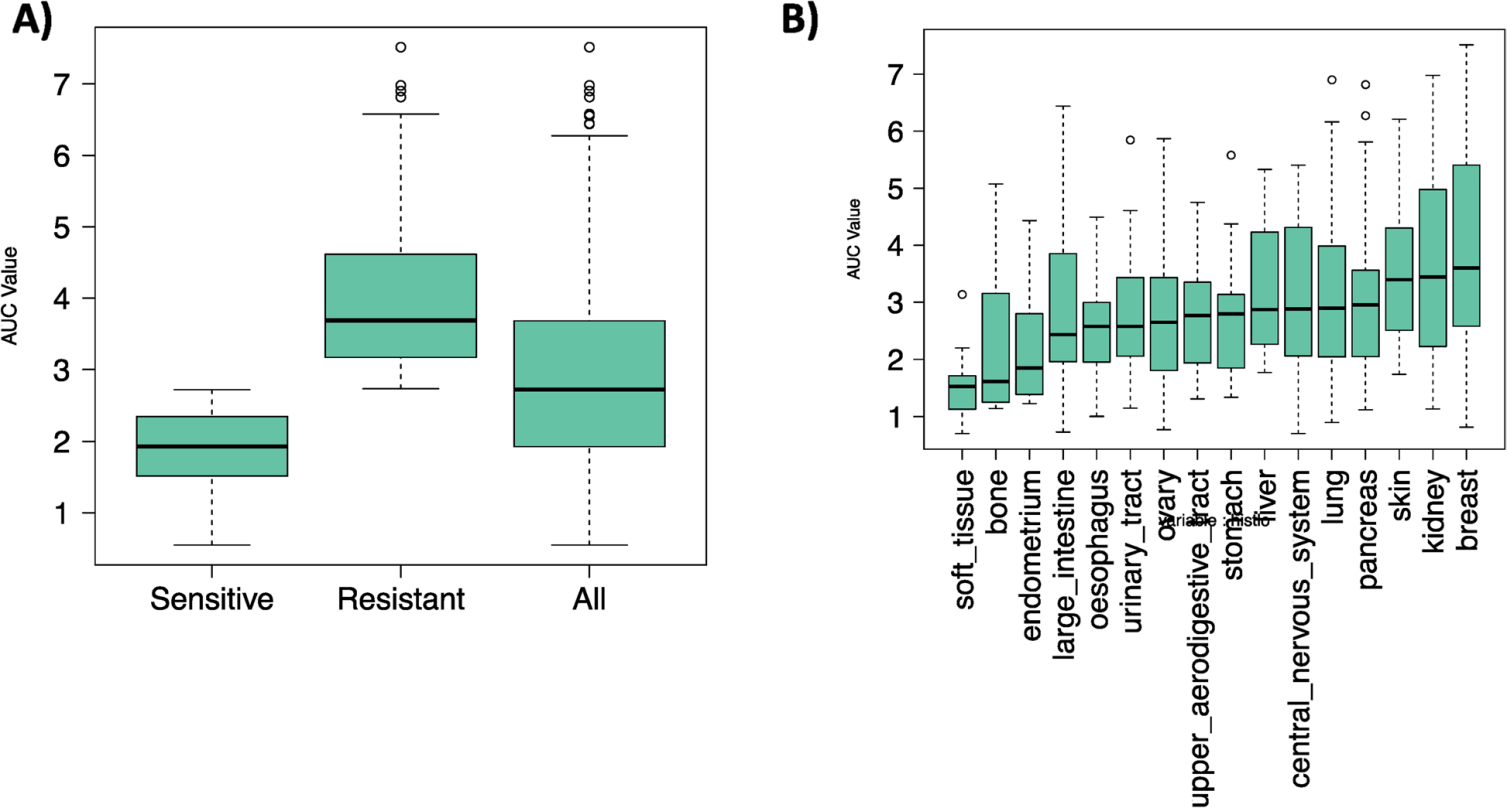
**A** Distribution of LQ model AUC values across all cell lines as well as the resistant and sensitive cell lines in the CL dataset. **B** Distribution of LQ model AUC values across various histologies (those cell lines with at least 10 per histology)

Using our *RadioGx* computational platform, we applied the LQ model to dose–response data for the cell lines available in the CL and NCI cohorts. The correlation between the computed AUC values with LQ model and the AUC assessed by the 9-day proliferative assay was found to be around 0.91 (*p* < e-16). Furthermore, we found the median value of AUC for all the cell lines in the CL cohort to be 2.71 (SD = 1.33). We then stratified the CL cohort into two groups—resistant and sensitive cell lines, based on the mean value of AUC. The median and standard deviation of AUC values for resistant and sensitive groups were found to be 3.68 (SD = 1.02) and 1.92 (SD = 0.53) respectively (Fig. 2A).

To assess the differences of radiation response distribution in AUC values across all tissue types in the largest dataset (CL cohort), we plotted the distribution of LQ model AUC values across various histologies (with a minimum number of 10 cell lines per histology). We observed that radiation response varied across tissues (Fig. 2B). We found that soft tissue and breast have the lowest and highest median AUC values, respectively.

### Comparison of radiation response indicators

**Fig. 3:**
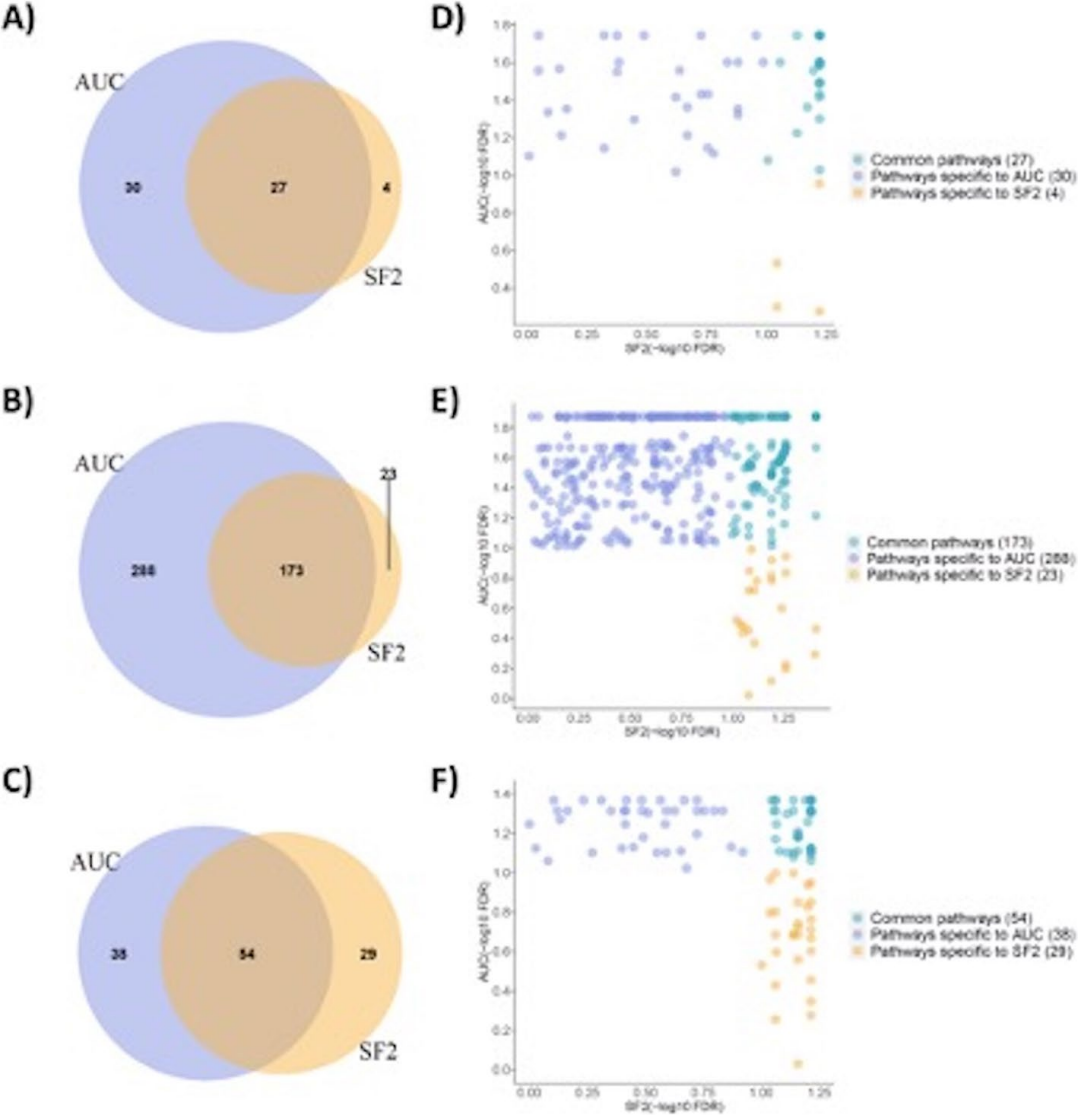
Comparison of biological processes underpinning radioresponse indicators, AUC and SF2. Panels **A**-**C** Venn diagrams representing pathways enriched with AUC and SF2 using KEGG, REACTOME and WIKIPATHWAYS databases, respectively. Panels **D**-**F** FDR for each biological pathway from **A**, **B**, **C** panels demonstrating greater levels of statistical significance among pathways specific to AUC compared to SF2

For carrying out any pre-clinical investigations along with the discovery of novel biomarkers, utilizing the robust radiation response indicator is crucial. To examine the biological pathways that were driving SF2 and AUC, we performed the pathway analysis on the largest available dataset, i.e., the CL dataset with 511 cell lines. Gene expression profiles were correlated with SF2 and/or AUC using the Spearman correlation. GSEA methodology was then applied on the ranked gene list based on the coefficient of correlation. All the results presented in this section were corrected for an FDR < 10% (Fig. 3).

Using the KEGG database (Fig. 3A and D), 57 molecular pathways were found to be enriched using AUC, out of which, 33 and 24 pathways were positively and negatively correlated with AUC, respectively. In a similar manner, using SF2, only 31 pathways were enriched, out of which, 19 were positively correlated with the SF2. There were 27 pathways that were commonly enriched between AUC and SF2. While 30 transcriptional pathways were specifically enriched with AUC, only 4 pathways were enriched specifically with SF2. Similarly, we have performed pathway analysis using the REACTOME database too (Fig. 3B and E). 461 biological pathways were enriched using AUC, out of which, 152 and 309 pathways were positively and negatively correlated with AUC, respectively. With SF2, only 196 pathways were enriched, out of which 81 and 115 were positively and negatively correlated. Among the enriched pathways, we found 173 of them to be common between SF2 and AUC. Moreover, 288 and 23 pathways were enriched specifically with AUC and SF2, respectively. Using the WIKIPATHWAYS database, 92 pathways were found to be enriched with AUC, out of which, 51 and 41 of them were positively and negatively enriched (Fig. 3C and F). While with SF2, we found 83 pathways to be enriched with 55 and 28 of them positively and negatively correlated with SF2. There were 54 transcriptional pathways that were commonly enriched between the two radioresponse indicators, AUC and SF2. While 38 of these pathways were specifically enriched with AUC, only 29 pathways were enriched specifically with SF2. Importantly, we found two groups of biologically enriched mechanisms, namely, cell cycle and repair pathways. It is well known that cell cycle post irradiation is known to determine radiation-induced cell death, and DNA repair is an important component for cell survival post irradiation [16]. Among these enriched pathways, cell cycle pathways were found to be negatively correlated with both SF2 and AUC, DNA damage and repair pathways are predominantly impaired in AUC. On the contrary, SF2 was associated with a more pronounced antioxidative response. We found an enrichment of Nrf2-related pathways, which are known to be activated in oxidative stress and promote cell protection and survival. Numerous works have shown that the activation of the Nrf2 biological pathway promotes radiation resistance through increased cyto-protection and cell growth [17–19].

To summarize our findings, we characterized the biological determinants underpinning SF2 and AUC, supporting the biological relevance of these transcriptional pathways in the realm of radiation therapy [16–19]. More importantly, we leveraged three pathway databases and found that AUC captures more biological processes compared to the point estimate, SF2. Furthermore, AUC was able to provide a more comprehensive characterization of the molecular processes underpinning radioresponse compared to SF2. Therefore, from our analyses, we conclude that AUC was able to capture more gene expression pathways that were correlated with radioresponse, compared to SF2. Altogether, our findings reveal that AUC is a better and a robust indicator of radiation response. As a result of these findings at the transcriptomic level, we exclusively used AUC as the radioresponse indicator to build the predictive models. Furthermore, our analyses demonstrated that cancer cells are guided by variable biological responses based on the radiation response indicator, which is a key determinant for biomarker discovery.

### Analysis of leading-edge genes

**Fig. 4:**
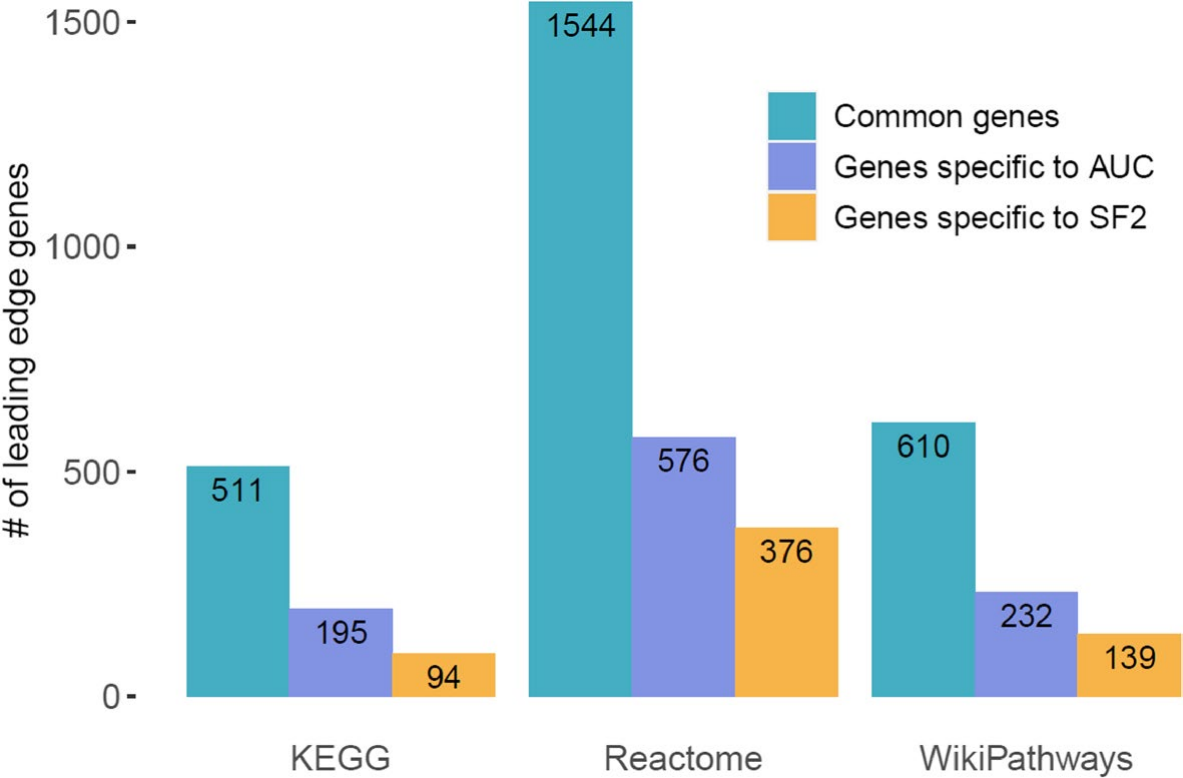
Comparison of leading-edge gene sets among commonly enriched pathways using the radiation response indicators, AUC and SF2, within each of the three pathway databases

To identify those genes that were driving the enrichment of biological pathways, we performed the leading-edge gene analysis. To achieve this, we focused on pathways that were commonly enriched between AUC and SF2 within each pathway database, separately. Leading-edge genes were extracted from 27, 173 and 54 common pathways that were enriched between AUC and SF2 in KEGG, REACTOME and WIKIPATHWAYS databases, respectively. The intersection of the unique leading-edge gene sets between AUC and SF2 is presented in Fig. 4. Despite the fact that there are a number of leading-edge genes that were overlapping across the pathway databases, there are a subset of genes that were specifically unique to AUC and SF2. These results suggest that the enrichment signal is driven by a different set of genes with the two radioresponse indicators, AUC and SF2. Additionally, this illustrates that cancer cells are driven by variable subsets of genes based on the radiation response indicator, which is crucial to build biomarkers.

### Model evaluation

**Fig. 5:**
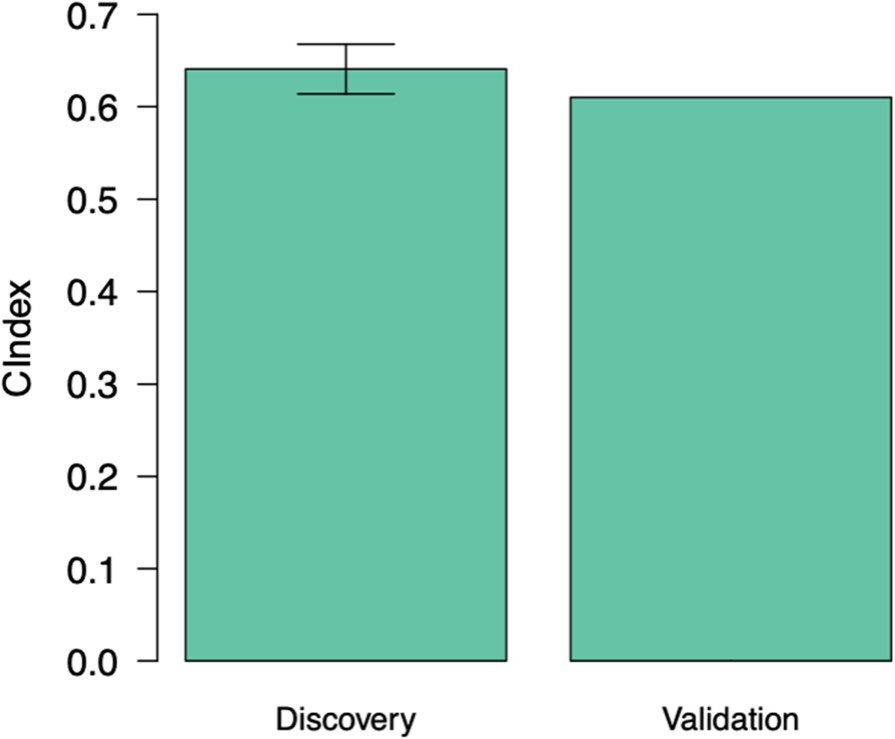
Model performance of genomic predictor of radiation sensitivity in the pre-validation and validation phases. The prediction performance of the predictive model assessed by concordance index between the predicted and observed AUC values. Predictions were averaged in 10 iterations of 10-fold cross-validation in the discovery dataset (CL). The error bars represent the 95% confidence interval of the performance computed during the 10 repetitions of cross-validation

In this section, we will present the results of the gene expression signature for predicting radiation sensitivity. We developed the genomic predictive model in the discovery dataset (CL) using a cross-validation framework consisting of 10 iterations of 10-fold cross-validations. In the 10-fold cross-validation approach, the multivariate regression model was developed using 90% of the total cell lines as a training set, leaving 10% of the cell lines as a test set. The gene expression profiles and AUC of all cell lines in the training set were used to identify features that were strongly associated with radiation response. To reduce the dimensionality of feature space, we selected those genes that were associated with radiation response. Then, the model’s performance was assessed using the concordance index in the pre-validation phase, which is presented in Fig. 5. The number of features that resulted in the highest concordance index was found to be 22. The gene signature can be found in the Supplementary file. We then used the set of best features and repeated 10 iterations in a 10-fold cross validation framework. The model yielded an average concordance index of 0.65 in the pre-validation phase. We further validated the performance of the genomic predictor in the NCI dataset, which is a fully independent validation cohort. This will enable us to examine if the developed gene expression signature can be generalizable to new datasets. On the validation cohort, the concordance index of the multivariate model was found to be 0.61**.**

## Discussion

To date, the paradigm of precision medicine has primarily been applied to pharmacogenomics, and little focus has been given to radiation oncology [4]. Radiotherapy is often used as a curative therapeutic intervention for early-stage curable cancers. The last few decades have witnessed an improvement in the survival rates of cancer patients with advancements in the physical precision of radiotherapy (RT) targeting of tumors. The clinical gains against these technological changes have been less impressive. Hence, to achieve a substantial gain in tumor control, therapeutic strategies have to be designed based on the genomic profiles of each patient, also known as the biological precision. With a rapid progress in high-throughput technologies and generation of sequencing data, it is now possible to leverage these diverse datasets and build molecular predictors of radiation response.

It is widely accepted that the next wave of clinical gains will be from designing biologically-guided radiation regimens. The availability of OMICS data has accelerated research towards developing data-driven OMICS-based biomarkers using gene expression profiles from in-vitro or cell line data [13, 20]. Several studies in the literature have developed radiation sensitivity gene signatures using cell data obtained from clonogenic survival assays, and an overview of these signatures can be found in a recent work by Manem et al. [11]. All of these studies built the radiosensitivity gene signatures using the NCI-60 panel or used SF2 as the response variable. These models demonstrated poor predictive performance on large-scale prospective validation datasets. Although not an exhaustive list, but, there are several reasons for the poor model performance, such as lack of assay standardization to generate dose–response data across labs, various statistical methods used to build these signatures resulting in reproducibility issues, and inadequate sample size, thereby evading their clinical translation [21, 22]. This highlights the need to develop robust and reproducible molecular predictors of radiation sensitivity for future interventional studies. Currently, there are no clinically approved genomic biomarkers predictive of radiation response. Furthermore, there is a lack of predictive biomarkers in the context of combination interventions, in which, radiotherapy is administered along with cytotoxic chemotherapeutic compounds and/or immunotherapy. Hence, there remains an unmet clinical need to develop robust and reproducible biomarkers of radiation response.

With this premise, we sought to address two objectives in this study, namely, *i)* compare the molecular determinants of the two radiation response indicators, SF2 and AUC using three pathway databases; and *ii)* build and validate the genomic predictor of radiation sensitivity using AUC as the response variable. To achieve the objectives of this work, we utilized two large-scale radiogenomics datasets consisting of 511 cancer cell lines (used as discovery cohort) and an external cohort of 60 cell lines (used as validation cohort). So far, none of the studies have utilized them to build and validate gene signatures. In addition, all of the studies in the literature have used SF2 as the radioresponse indicator, although, mathematically, integral under the radiobiological curve captures more biological processes, as presented in this study. We performed pathway analysis using the GSEA method and demonstrated that AUC captures more molecular processes compared to SF2. We found this finding to be agnostic to the pathway database. Despite radiation conserves numerous biological pathways, unique biological signals were enriched based on the radiation response indicator, which is a crucial determinant for building biomarkers. These findings will further facilitate a potential understanding on the biological mechanisms driving radiation sensitivity at the transcriptomic level. As a result of these findings, we have used AUC as the radioresponse indicator to build the predictive models. To build the molecular predictor, we implemented a multivariate regression model with AUC as the radiation response variable. The CL dataset was used as the discovery cohort (*n* = 511 cancer cell lines) and the NCI dataset was used as the validation cohort (*n* = 60 cancer cell lines). We found the concordance index of 0.65 in the discovery dataset and 0.61 in the validation dataset. It should be noted that the model performance is in line with other cell line-derived drug response studies in the literature. One of the major reasons for the model performance to be around 65% can be attributed to the complexity of the experimental design and assays used, and the tradeoff between various experimental parameters to enable high throughput collection of data makes the noise unavoidable from these generated data. More advanced statistical approaches should be employed to correct for these noisy observations to build robust predictive biomarkers, which is a subject of ongoing investigation.

This study holds great promise to generate more testable biological hypotheses, for instance, SF2 vs AUC indicators in a pre-clinical setting along with the development of novel biomarkers using the integral of the dose–response curve. Our work also represents a significant step towards individualizing radiation dosimetry with the integral of the dose–response curve to individual patients and not abiding to the one- size-fits all philosophy that is currently employed in routine clinical care. Importantly, the integration of clinical and OMICS features in the predictive model, will help the physicians to design optimal radiation treatment plans that can maximize the tumor control and minimize the toxicities. While this study is promising, we do acknowledge the limitations. Firstly, the complex experimental assays with non-standard distributions may potentially lead to non-spurious associations between genomics features and radioresponse indicators. Secondly, it is possible that cell lines may have evolved under different conditions temporally and accumulated genomic alterations that were re culture-dependent. Thirdly, these cell cultures lack the tumor microenvironment structure such as stroma and immune cells, which play a major role in radiation response. Hence, it is pertinent to address these issues in future works by using rigorous quality controls in experiments.

Rigorous model testing is required before adopting the genomic predictors in clinical practice [23]. To demonstrate the robustness and generalizability, gene signatures have to be validated on several external datasets as well as on prospective cohorts, if available. To summarize, we envision that the developed gene signature of radiation sensitivity based on the integral of the dose–response curve has the enormous potential to personalize RT and improve the treatment outcomes. This could eventually have a huge impact in decision-making landscape of precision radiation oncology.

### Supplementary Information

**Additional file 1.**

## Data Availability

All the data is publicly available and can be downloaded from the *RadioGx* platform: https://bioconductor.org/packages/release/bioc/html/RadioGx.html.

## References

[1] Moll M, Herrmann H, Zaharie A, Goldner G (2022). Advancements in the radiooncological treatment of high-risk prostate cancer: a quarter century of achievements. Radiol Oncol.

[2] Parisi S, Ferini G, Lillo S, Brogna A, Chillari F, Ferrantelli G, Settineri N, Santacaterina A, Platania A, Leotta S, Casablanca G, Russo A, Pontoriero A, Adamo V, Minutoli F, Bottari A, Cacciola A, Pergolizzi S (2023). Stereotactic boost on residual disease after external-beam irradiation in clinical stage III non-small cell lung cancer: mature results of stereotactic body radiation therapy post radiation therapy (SBRTpostRT) study. Radiol Med.

[3] Clark CA, Yang ES (2023). Therapeutic targeting of DNA damage repair in the era of precision oncology and immune checkpoint inhibitors. J Immunother Precis Oncol.

[4] Baumann M, Krause M, Overgaard J, Debus J, Bentzen SM, Daartz J, Richter C, Zips D, Bortfeld T (2016). Radiation oncology in the era of precision medicine. Nat Rev Cancer.

[5] E.G.C. Troost. Image-guided high-precision radiotherapy. Berlin: Springer Nature; 2022.

[6] Grass GD, Mills MN, Scott JG, Eschrich S, Torres-Roca JF (2019). Genomics and radiomics: tools to see the unseen to personalize radiation therapy. Appl Radia Ontcol.

[7] Manem VSK, Dhawan A, Kohandel M, Sivaloganathan S (2014). Efficacy of dose escalation on TCP, recurrence and second cancer risks: a mathematical study. Br J Radiol.

[8] Manem VSK, Dhawan A (2018). Modelling recurrence and second cancer risks induced by proton therapy. Math Med Biol.

[9] Manem VSK, Grassberger C, Paganetti H (2017). Predicting organ-specific risk interactions between radiation and chemotherapy in secondary cancer survivors. Cancers.

[10] Bentzen SM, Overgaard J (1994). Patient-to-patient variability in the expression of radiation-induced normal tissue injury. Semin Radiat Oncol.

[11] Manem VS, Dhawan A (2019). a database of oxic and hypoxic radiation response gene signatures and their utility in pre-clinical research. Br J Radiol.

[12] Yard BD, Adams DJ, Chie EK, Tamayo P, Battaglia JS, Gopal P, Rogacki K, Pearson BE, Phillips J, Raymond DP, Pennell NA, Almeida F, Cheah JH, Clemons PA, Shamji A, Peacock CD, Schreiber SL, Hammerman PS, Abazeed ME (2016). A genetic basis for the variation in the vulnerability of cancer to DNA damage. Nat Commun.

[13] Manem VS, Lambie M, Smith I, Smirnov P, Kofia V, Freeman M, Koritzinsky M, Abazeed ME, Haibe-Kains B, Bratman SV (2019). Modeling cellular response in large-scale radiogenomic databases to advance precision radiotherapy. Cancer Res.

[14] Subramanian A, Tamayo P, Mootha VK, Mukherjee S, Ebert BL, Gillette MA, Paulovich A, Pomeroy SL, Golub TR, Lander ES, Mesirov JP (2005). Gene set enrichment analysis: a knowledge-based approach for interpreting genome-wide expression profiles. Proc Natl Acad Sci U S A.

[15] Väremo L, Nielsen J, Nookaew I (2013). Enriching the gene set analysis of genome-wide data by incorporating directionality of gene expression and combining statistical hypotheses and methods. Nucleic Acids Res.

[16] Deng S, Vlatkovic T, Li M, Zhan T, Veldwijk MR, Herskind C (2022). Targeting the DNA damage response and DNA repair pathways to enhance radiosensitivity in colorectal cancer. Cancers.

[17] McDonald JT, Kim K, Norris AJ, Vlashi E, Phillips TM, Lagadec C (2010). Ionizing radiation activates the Nrf2 antioxidant response. Cancer Res.

[18] Ohta T, Iijima K, Miyamoto M, Nakahara I, Tanaka H, Ohtsuji M (2008). Loss of Keap1 function activates Nrf2 and provides advantages for lung cancer cell growth. Cancer Res.

[19] Zimta AA, Cenariu D, Irimie A, Magdo L, Nabavi SM, Atanasov AG (2019). The role of Nrf2 activity in cancer development and progression. Cancers.

[20] Manem VSK (2021). Development and validation of genomic predictors of radiation sensitivity using preclinical data. BMC Cancer.

[21] Baine MJ, Lin C (2017). Genome-based modeling for adjusting radiotherapy dose (GARD)-a significant step toward the future of personalized radiation therapy, Transl. Cancer Res.

[22] Bratman SV, Milosevic MF, Liu FF, Haibe-Kains B (2017). Genomic biomarkers for precision radiation medicine. Lancet Oncol.

[23] McShane LM, Cavenagh MM, Lively TG, Eberhard DA, Bigbee WL, Williams PM, Mesirov JP, Polley MYC, Kim KY, Tricoli JV, Taylor JMG, Shuman DJ, Simon RM, Doroshow JH, Conley BA (2013). Criteria for the use of omics-based predictors in clinical trials: explanation and elaboration. BMC Med.

